# Leptomeningeal Carcinomatosis Arising 29 Years After Gastrectomy: A Report of a Rare Autopsy Case

**DOI:** 10.7759/cureus.107630

**Published:** 2026-04-24

**Authors:** Kouki Mizuno, Naohisa Ueda, Katsuya Abe, Katsuo Kimura, Fumiaki Tanaka

**Affiliations:** 1 Neurology, Yokohama City University Medical Center, Yokohama, JPN; 2 Neurology and Stroke Medicine, Yokohama City University Graduate School of Medicine, Yokohama, JPN

**Keywords:** gastric cancer (gc), late recurrence, leptomeningeal carcinomatosis (lmc), signet ring cell carcinoma, tumor dormancy

## Abstract

Leptomeningeal carcinomatosis (LMC) is extremely rare in gastric cancer, as is recurrence >10 years after gastrectomy. Herein, we report an autopsy case of LMC that developed 29 years after gastrectomy for gastric signet ring cell carcinoma. A 74-year-old man presented with multiple progressive cranial neuropathies involving cranial nerves III, V, VI, VII, IX, X, and XII. CSF analysis revealed pleocytosis and markedly elevated protein levels; however, cytological examination of five consecutive samples was negative for malignant cells. Multiplex PCR analysis excluded infectious etiologies. MRI demonstrated contrast enhancement of cranial nerves III, V, VII, and XII. Despite antiviral, antituberculous, and corticosteroid therapy, the patient’s condition deteriorated, and he ultimately died three months after symptom onset. Autopsy revealed extensive infiltration of signet ring cell carcinoma in the leptomeninges and cranial nerves. Immunohistochemical analysis of this tissue revealed cytokeratin 7 positivity and cytokeratin 20 negativity, identical to the gastric carcinoma resected 29 years earlier. No recurrence was observed in the remnant stomach or other organs. This case suggests reactivation of microscopic leptomeningeal metastases following an exceptionally prolonged period of tumor dormancy. Signet ring cell carcinoma is thought to be prone to dormancy owing to its weak intercellular adhesion, leading to reduced proliferative signaling and activation of stress response pathways. This experience suggests that LMC should be considered when meningitis-like neurological symptoms develop, even in patients who remain recurrence-free for decades postoperatively.

## Introduction

Leptomeningeal carcinomatosis (LMC) is a rare and fatal malignancy characterized by the dissemination of malignant cells from solid tumors or hematological malignancies to the pia mater, arachnoid mater, and subarachnoid space. Patients present with diverse neurological manifestations, including headache, cranial nerve palsy, and impaired consciousness [1]. LMC occurs in approximately 3-5% of patients with malignancies and is most commonly associated with lung cancer, breast cancer, and malignant melanoma [2]. In contrast, LMC originating from gastric cancer is exceedingly rare, with a reported incidence of only 0.06-0.24% [3,4]. Moreover, gastric cancer recurrence typically occurs within two years postoperatively, with this timeline accounting for approximately 79% of cases [5]. Conversely, recurrence >10 years after gastrectomy has been reported in only 0.9% of patients [6]. Herein, we describe an autopsy case of LMC that developed 29 years following gastrectomy for gastric cancer, representing an extremely rare pattern of late recurrence.

## Case presentation

The patient was a 74-year-old man who presented with facial motor and sensory impairments. One month before admission, he had developed bilateral upper facial pain that gradually extended to the perioral region, followed by difficulty in opening his mouth two weeks later. He had lost approximately 5 kg over the preceding six months, with a body weight of 44.3 kg. His medical history included a subtotal gastrectomy with Billroth II reconstruction for gastric signet ring cell carcinoma 29 years prior, with no intervening recurrence. Two years before admission, he experienced herpes zoster in the trigeminal nerve distribution, which resolved without sequelae. Comorbidities included hypertension, diabetes mellitus, and lumbar spinal canal stenosis.

On examination, the patient was alert and had stable vital signs. Ocular motor examination revealed mild limitation of abduction and elevation in the right eye and severe limitation of abduction with moderate limitation of adduction in the left eye. Neurological examination revealed tingling dysesthesia over the entire face and bilateral masseter muscle weakness with restricted mouth opening, dysarthria, and rightward tongue deviation. These findings indicated multiple cranial neuropathies involving the oculomotor, trigeminal, abducens, facial, glossopharyngeal, vagus, and hypoglossal nerves. Limb strength and sensation were preserved; however, dysmetria was present in the upper limbs, while ataxia with decomposition of movement was observed in the lower limbs. Neurological examination findings raised the possibility of several etiologies, including infectious diseases of the brainstem such as tuberculosis, inflammatory disorders including sarcoidosis, neoplastic involvement of the brainstem, and brainstem vascular disorders.

Laboratory tests revealed mildly elevated blood urea nitrogen (25 mg/dL), hyponatremia (122 mEq/L), mild hyperkalemia (5.5 mEq/L), and a slight increase in C-reactive protein (0.4 mg/dL). Immunological studies revealed no autoantibodies except for a mildly elevated rheumatoid factor (16.7 IU/mL) (Table 1).

**Table 1:** Results of blood tests conducted on admission ANCA, anti-neutrophil cytoplasmic antibody; dsDNA, double-stranded DNA; SS-A, Sjögren’s syndrome-related antigen A; SS-B, Sjögren’s syndrome-related antigen B

Immunology panel	Results	Reference range
IgG, mg/dL	1180	861-1747
IgG4, mg/dL	18.9	11-121
IgA, mg/dL	151	93-393
IgM, mg/dL	43	33-183
Rheumatoid factor, IU/dL	16.7	<15.0
Antinuclear antibody titer	<40	<40
Anti-dsDNA antibody, IU/mL	<2.0	<6
Anti-SS-A antibody, U/mL	<1.0	<10.0
Anti-SS-B antibody, U/mL	<1.0	<10.0
Anti-glutamic acid decarboxylase antibody, U/mL	<5.0	<5.0
Anti-aquaporin 4 antibody, U/mL	<1.5	<3.0
Proteinase 3-ANCA, U/mL	<1.0	<3.5
Myeloperoxidase-ANCA, U/mL	<1.0	<3.5
Tumor markers	Results	Reference range
Carcinoembryonic antigen, ng/mL	4.9	<5.0
Carbohydrate antigen 19-9, U/mL	18	<37
Prostate-specific antigen, ng/ml	4.87	<4
Neuron-specific enolase, ng/mL	11	<16.3
Soluble interleukin-2 receptor, U/mL	431	122.0-496.0
Cytokeratin 19 fragment, ng/mL	7.9	<2.8
Thymidine kinase, U/L	6.7	<7.5
Squamous cell carcinoma antigen, U/L	0.9	0.6-2.5

Serological screening for herpes simplex virus (HSV), varicella zoster virus (VZV), *Mycobacterium tuberculosis*, syphilis, HIV, and fungal infections yielded negative results. Among the tumor markers analyzed, prostate-specific antigen levels were mildly elevated (4.87 ng/mL) (Table 1). CSF analysis demonstrated mononuclear pleocytosis (65 cells/µL), markedly elevated protein levels (957 mg/dL), normal glucose levels (86 mg/dL), increased IgG (concentration: 229 mg/dL), and a mildly elevated IgG index (0.82) (Table 2). Multiplex PCR analysis using the FilmArray® Meningitis and Encephalitis Panel (BioFire Diagnostics, LLC, Salt Lake City, UT, USA) was negative for HSV types 1 and 2, VZV, human herpesvirus 6, human parechovirus, cytomegalovirus, enterovirus, *Escherichia coli *K1, *Haemophilus influenzae*, *Listeria monocytogenes*, *Neisseria meningitidis*, *Streptococcus agalactiae*, *Streptococcus pneumoniae*, *Cryptococcus neoformans*, and *Cryptococcus gattii*. CSF cytology was performed five times, and no malignant cells were detected in any of the samples (Table 2).

**Table 2:** Results of CSF tests conducted at admission HSV, herpes simplex virus

CSF parameter	Results	Reference range
Cells, /μL	65	0-5
Protein, mg/dL	957	10-40
Glucose, mg/dL	86	50-75
IgG, mg/dL	229	1.46-3.36
IgG index	0.82	0.28-0.66
Oligoclonal bands	Negative	Negative
Multiplex PCR analysis (FilmArray Meningitis/Encephalitis Panel); HSV type 1, HSV type 2, varicella-zoster virus, human herpesvirus 6, human parechovirus, cytomegalovirus, enterovirus, *Escherichia coli *K1, *Haemophilus influenzae*, *Listeria monocytogenes*, *Neisseria meningitidis*, *Streptococcus agalactiae*, *Streptococcus pneumoniae*, *Cryptococcus neoformans*, and *Cryptococcus gattii*	All negative	All negative
Cytology (×5)	No malignant cells	No malignant cells

**Figure 1:**
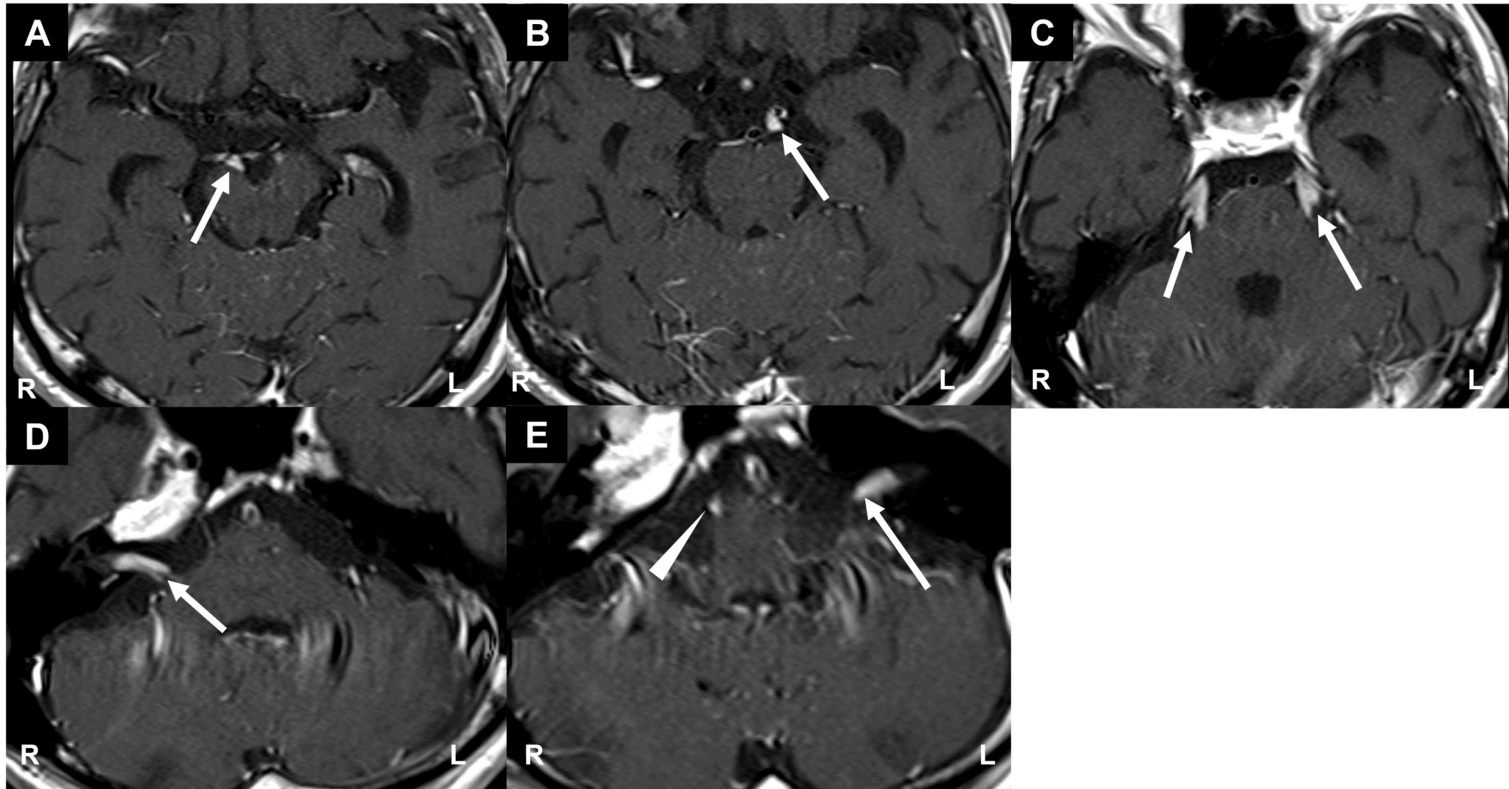
Contrast-enhanced T1-weighted MRI showing enhancement of bilateral oculomotor nerves (A, B; arrows), bilateral trigeminal nerves (C; arrows), bilateral facial nerves (D, E; arrows), and the right hypoglossal nerve (E; arrowhead)

Contrast-enhanced T1-weighted MRI demonstrated enhancement of the bilateral oculomotor, trigeminal, and facial nerves, as well as the right hypoglossal nerve (Figure 1). Chest and abdominal CT revealed no apparent neoplastic lesions, and PET-CT revealed no definite abnormal tracer uptake. 

Although VZV was not detected in the CSF, aseptic meningitis with multiple cranial neuropathies due to VZV reactivation was suspected based on the patient’s clinical course and prior trigeminal herpes zoster. Therefore, IV acyclovir was initiated. However, tuberculous meningitis and autoimmune meningitis, including neurosarcoidosis and hypertrophic pachymeningitis, could not be excluded. Accordingly, antituberculous therapy was administered together with corticosteroids (IV therapy thrice per week for three weeks followed by oral therapy). Despite these treatments, the patient’s condition failed to improve, consciousness progressively deteriorated, and he developed multiple organ failure, resulting in death after three months.

**Figure 2:**
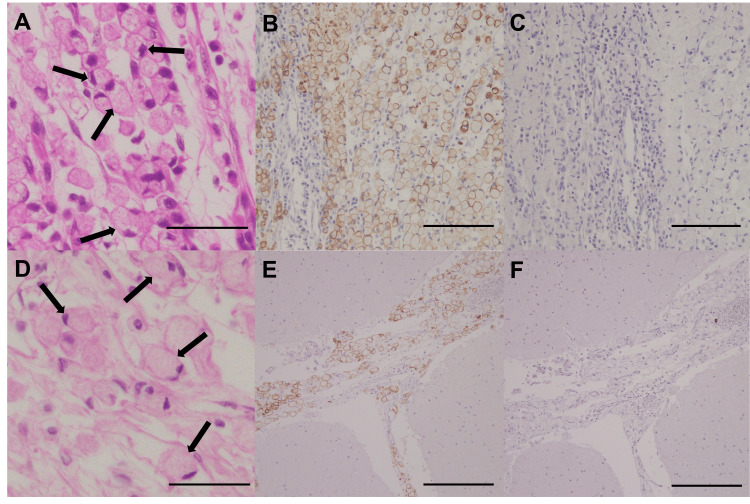
Histopathological findings of the gastric tissue resected 29 years prior and the meningeal tissue obtained at autopsy Histopathological findings of the gastric (A-C) and meningeal (D-F) tissues. Hematoxylin and eosin staining (A, D) showing signet-ring cell carcinoma in the gastric and meningeal tissues; arrows indicate signet-ring-like cells with eccentrically located nuclei. Immunohistochemical staining showing CK7 positivity (B, E) and CK20 negativity (C, F) in both tissues. Scale bars: A, D, 50 µm; B, C, 100 µm; E, F, 200 µm. CK7, cytokeratin 7; CK20, cytokeratin 20

Autopsy revealed diffuse infiltration of atypical cells with eccentrically located nuclei, consistent with the signet ring cell carcinoma resected 29 years earlier, throughout the cranial nerves and meninges of the cerebrum, cerebellum, and spinal cord. Immunohistochemical staining for cytokeratin 7 (CK7) and cytokeratin 20 (CK20) demonstrated that both the primary gastric cancer and meningeal lesions were CK7-positive and CK20-negative, respectively, showing an identical staining pattern consistent with signet-ring cell carcinoma (Figure 2). Based on these findings, the patient was definitively diagnosed with LMC due to the recurrence of gastric signet ring cell carcinoma. No atypical cells were identified in the other organs, including the remnant stomach.

## Discussion

This case represents an exceedingly rare autopsy-confirmed instance of LMC that recurred 29 years following surgical resection of gastric cancer, representing an exceptionally long disease-free interval. Although lung and breast cancers are more prone to LMC than gastric cancer, postoperative recurrence in the form of LMC has been reported only rarely; the longest documented disease-free interval was 17 years for lung cancer [7] and 10 years for breast cancer [8]. Recurrence of gastric cancer > 20 years after surgery is extremely uncommon; however, our literature review identified three similar cases (Table 3) [9-11]. All reported patients were male, and signet ring cell carcinoma was identified either in the primary tumor or in metastatic sites, such as the peritoneum, bone, or bone marrow. Among the reported cases of gastric cancer recurrence presenting as LMC, the longest previously documented interval between surgery and recurrence was four years [12], underscoring the extraordinary nature of the present case. In our patient, no residual or metastatic tumor was identified in the remnant stomach or other organs during autopsy. This finding indicates that microscopic metastases to the central nervous system may have occurred preoperatively or in the early postoperative period, followed by a prolonged dormant state before the tumor cells eventually acquired proliferative capacity and manifested clinically as LMC.

**Table 3:** Overview of previously reported cases of late-onset recurrence of gastric cancer over 20 years after surgery

No.	Author	Age	Sex	Histological type of the primary tumor	Interval after surgery	Recurrence site	Histological type of the metastatic tumor
1	Okugawa et al. (2010) [8]	61	M	Tubular adenocarcinoma with partial signet ring cell carcinoma	20 years	Peritoneum	Tubular adenocarcinoma with signet-ring cell carcinoma
2	Blanchette et al. (2013) [9]	66	M	Adenocarcinoma	22 years	Bone and bone marrow	Signet-ring cell carcinoma
3	Rodrigues et al. (2024) [10]	73	M	Signet-ring cell carcinoma	20 years	Peritoneum and bone	Adenocarcinoma
4	Present case	74	M	Signet-ring cell carcinoma	29 years	Meninges and cranial nerves	Signet-ring cell carcinoma

Late recurrence of gastric cancer cannot be predicted by established prognostic factors for early recurrence, such as tumor stage, tumor size, depth of invasion, lymphatic invasion, lymph node metastasis, or surgical margin status [10,13]. Consequently, tumor dormancy has been strongly implicated as a key mechanism underlying late recurrence [6,9,10]. Tumor dormancy is defined as a biological state in which disseminated tumor cells persist for long periods in a dynamic equilibrium between proliferation and apoptosis, ultimately being reactivated after a prolonged latent period [14]. This concept is widely accepted as the most plausible explanation for sudden recurrence after an extended disease-free interval. While tumor dormancy provides a plausible explanation for the exceptionally late recurrence observed in this case, it cannot be directly proven, and alternative mechanisms cannot be completely excluded.

Among the various classified gastric cancers, signet ring cell carcinoma appears to be particularly prone to tumor dormancy. Indeed, most reported cases of gastric cancer recurrence occurring > 20 years after surgery involve this histological subtype (Table 3) [9-11], as was observed in the present case. Signet-ring cell carcinoma is characterized by reduced cell-cell adhesion due to altered expression of epithelial-mesenchymal transition-related genes and the decreased expression of adhesion molecules, resulting in a diffuse infiltrative growth pattern [15]. Failure to establish stable adhesion with neighboring cells leads to the attenuation of proliferative signaling and activation of stress-response pathways, including the p38 MAPK pathway, consequently predisposing tumor cells to enter a G0-like dormant state [16]. These dormant cells retain stem cell-like properties and the capacity to reproliferate [17]. The reactivation of dormant tumor cells may be influenced by changes in the host immune environment. Impairment of immune surveillance due to aging, chronic inflammation, infection, or systemic physiological changes may shift the host-tumor interaction from an equilibrium phase to an escape phase, consequently triggering tumor reactivation [6,9,10]. Although the precise trigger for tumor reactivation in the present case remains unclear, the preceding weight loss may have been associated with alterations in immune function.

Antemortem diagnosis of LMC remains a challenge in some cases. In the present patient, malignant cells were not detected despite five CSF cytological examinations. Further, no definitive neoplastic lesions were identified on imaging studies, thus precluding a diagnosis during life. The sensitivity of CSF cytology is reported to be approximately 50% on initial examination; therefore, repeated testing is often recommended [1]. Nevertheless, approximately 10% of LMC cases remain negative even after multiple examinations [18], while up to 40% of autopsy-confirmed LMC cases show negative CSF cytology results [1]. Accordingly, diagnostic methods with higher sensitivity than conventional CSF cytology are urgently needed. Recent studies have demonstrated that liquid biopsy offers superior sensitivity compared with cytology [19,20] and may represent a promising diagnostic approach for detecting rare malignancies, including gastric signet ring cell carcinoma.

## Conclusions

Overall, this case represents an extremely rare autopsy-confirmed instance of LMC that recurred after an exceptionally prolonged tumor dormancy period of 29 years following gastric cancer surgery. Even among patients with solid tumors who have remained disease-free for decades after surgery, LMC should be considered when meningitis-like neurological symptoms emerge, even if imaging studies and repeat CSF examinations are negative.
